# Are cancer patients better off if they participate in clinical trials? A mixed methods study

**DOI:** 10.1186/s12885-020-06916-z

**Published:** 2020-05-08

**Authors:** Zandra Engelbak Nielsen, Stefan Eriksson, Laurine Bente Schram Harsløf, Suzanne Petri, Gert Helgesson, Margrete Mangset, Tove E. Godskesen

**Affiliations:** 1 Department of Oncology, Copenhagen University Hospital, Copenhagen, Section 5073, Rigshospitalet, Blegdamsvej 9, 2100 Copenhagen, Denmark; 2 Centre for Research Ethics & Bioethics, Uppsala University, Box 564, 751 22, Uppsala, Sweden; 3 Stockholm Centre for Healthcare Ethics (CHE), LIME, Karolinska Institutet, 171 77 Stockholm, Sweden; 4 Department of Geriatric Medicine, Oslo University Hospital, Kirkeveien 166, Bygg 20, 0450 Oslo, Norway; 5 Department of Health Care Sciences, Ersta Sköndal Bräcke University College, Box 11189, 100 61 Stockholm, Sweden

**Keywords:** Pharmacological clinical trials, Physicians, Nurses, Neoplasms, Outcomes, Survival, Mixed methods, Qualitative, Literature review

## Abstract

**Background:**

Research and cancer care are closely intertwined; however, it is not clear whether physicians and nurses believe that clinical trials offer the best treatment for patients and, if so, whether this belief is justified. The aim of this study was therefore: (i) to explore how physicians and nurses perceive the benefits of clinical trial participation compared with standard care and (ii) whether it is justified to claim that clinical trial participation improves outcomes for cancer patients.

**Methods:**

A mixed methods approach was used employing semi-structured interviews with 57 physicians and nurses in oncology and haematology and a literature review of the evidence for trial superiority, i.e. the idea that receiving treatment in a clinical trial leads to a better outcome compared with standard care. Inductive thematic analysis was used to examine the interview data. A literature review comprising nine articles was conducted according to a conceptual framework developed by Peppercorn et al. and evaluated recent evidence on trial superiority.

**Results:**

Our findings show that many physicians and nurses make claims supporting trial superiority, however very little evidence is available in the literature comparing outcomes for trial participants and non-participants that supports their assertions.

**Conclusions:**

Despite the recent rapid development and use of targeted therapy and immunotherapy, we find no support for trial participation to provide better outcomes for cancer patients than standard care. Hence, our present results are in line with previous results from Peppercorn et al. A weaker version of the superiority claim is that even if a trial does not bring about a direct positive effect, it brings about indirect positive effects. However, as the value of such indirect effects is dependent on the individual’s specific circumstances and preferences, their existence cannot establish the general claim that treatment in trials is superior. Belief in trial superiority is therefore unfounded. Hence, if such beliefs are communicated to patients in a trial recruitment context, it would provide misleading information. Instead emphasis should be on patients volunteering to give an altruistic contribution to the furthering of knowledge and to the potential benefit of future patients.

## Background

**Fig. 1:**
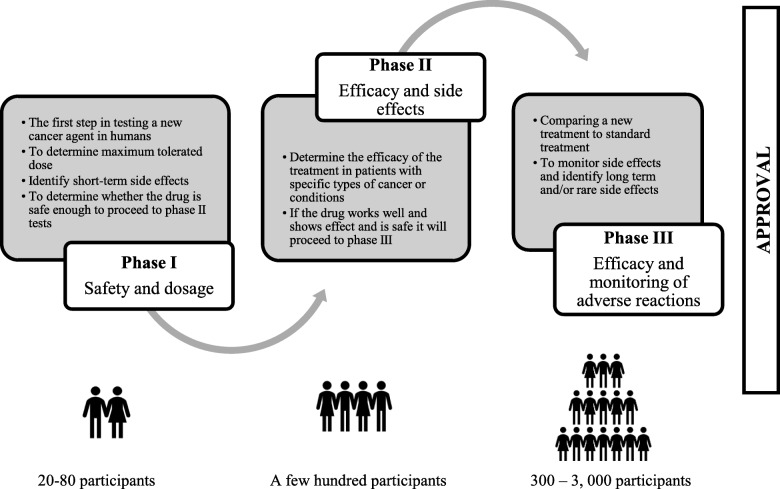
Characteristics of clinical trial phases according to U.S. Food & Drug Administration, FDA (https://www.fda.gov)

According to the World Health Organization nearly one in six deaths is due to cancer [1]. As such, interest in innovative cancer research and treatment breakthroughs is high [2–4]. There is a concern, however, that the generally modest incremental advances taking place are over-interpreted, particularly in oncology [5–7]. A study conducted by the Biotechnology Innovation Organization [8] on clinical development success rates between 2006 and 2015 showed that only a small number of drugs successfully make it from phase I trials to medical practice. Among oncology therapies in phase I, the probability of later gaining approval was only 5%. (For significance and difference between trial phases, see Fig. 1).

Why do cancer studies with such uncertain outcomes create so much excitement? Part of the explanation might lie in the way they are presented to the public. The lay press uses an abundance of superlatives to characterise breakthroughs. Abola et al. [9] studied news articles and showed that they used superlatives to describe about half of the cancer drugs approved by the U.S. Food & Drug Administration and half of the unapproved ones. Superlatives were also used to describe therapeutic cancer vaccines with low response rates, drugs that do not show overall survival benefits, and drugs untested on humans. An earlier study done by our research group looked at online descriptions of phase I trials that told prospective study participants and their physicians about the trials and found that the descriptions were sometimes misleading and provided almost no information about possible adverse effects or the disadvantages of study participation [10]. These and similar studies highlight that research often is presented with a strong focus on positive outcomes [11–13].

In oncology and haematology, research and standard care are closely intertwined. The associated blurring of boundaries between treatment and research may result in a stance that the best treatment for cancer always is provided within the context of a clinical trial. Peppercorn et al. [14] cited several typical examples of oncologists who believe that patients in trials have better outcomes than those not in a trial. In previous studies we found that this is an important issue for physicians and nurses who experience ethical challenges when clinical care and research are intertwined [15, 16]. Physicians and nurses found that a most difficult challenge was end-of-life patients eager to participate in all kinds of cutting-edge drug trials, often with an unrealistic hope for possible gain. The patients sometimes had high expectation concerning benefits [17–22], or believed the trials were miracles waiting to happen [23]. Though questionable, many physicians found it important to uphold hope and to support a belief in clinical trials because it can benefit patients and relatives. Nurses were often concerned about patients’ level of unrealistic hope and about their well-being sometimes becoming secondary to research participation. They sometimes wondered if palliative care would be more beneficial than participating in research [15]. In other words, belief in trial superiority might have costs, but if the belief is warranted, then disregarding it would have even worse consequences. As Peppercorn et al. [14] note, trial superiority would provide the basis to push much more strongly for enrollment of patients currently receiving sub-standard care and to enhance efforts to remove barriers to trial participation.

It is indeed imperative to ask whether there is a basis for the claim that patients usually, or as a rule, will have better outcomes by participating in a trial compared with not participating. Peppercorn et al. [14] reviewed primary data and compared outcomes between patients in and outside trials to examine the evidence. However, they found little high quality evidence supporting better outcomes. The researchers concluded that, “cancer patients should be encouraged to enrol in clinical trials on the basis of trials’ unquestioned role in improving treatment for future patients”, rather than because of the hype and misplaced hope. There is a need to re-examine, also partly in a new context, whether physicians and nurses really do assume that participation in pharmacology trials (hereafter clinical trials) benefit patients more than treatment with standard care – and whether they are justified in this belief. The aims of this study are to explore (i) how physicians and nurses perceive the benefits of clinical trial participation compared with standard care and (ii) whether it is justified to claim that clinical trial participation improves outcomes for cancer patients. The research questions are:

1. How do physicians and nurses perceive the benefits of clinical trial participation compared with standard care? (explorative/qualitative)
2. Does clinical trial participation provide better outcomes for cancer patients? (confirmatory/quantitative)

## Methods

### Design

#### A mixed methods approach

A mixed methods approach was chosen because it provides complementary data suitable for exploratory studies [24]. In accordance with O’Cathain et al. [25], we “followed a thread”. This approach involves collecting several sets of data and analysing them separately, and then combining them to reach a deeper understanding of the issue studied. You pick one issue, question or finding from one such research component and follow it across other components. In this study, interviews with physicians and nurses indicated a widespread belief that clinical trials provide the best treatment for cancer. This finding from the interview component was then analysed more closely, generating an additional question: Does clinical trial participation really provide better outcomes for cancer patients? We then explored this question further through a second component, a literature review. The final stage involved following the thread back to the interview data to further discuss and normatively deliberate about the qualitative findings in light of the literature review results.

In line with a mixed methods approach, our study comprised two parts. Part I is empirical and consists of semi-structured interviews, while Part II comprises a literature review to provide a comprehensive summary of current evidence relevant to the question of whether clinical trials are superior to standard care.

The empirical data were collected as part of a larger project (briefly summarised below) that is described in detail elsewhere, including recruitment and data collection [15, 16]. We followed the Standards for Reporting Qualitative Research (SRQR) in methods, results and discussion to improve the quality and transparency [26].

#### Part I: qualitative interviews

##### Selection and recruitment of interview participants

The informants were recruited at two university hospitals in Denmark and Sweden at three wards specialising in oncology and haematology. The 57 participants, most of them women, comprised 26 physicians and 31 nurses. The participants varied considerably in terms of age, years of practice and the clinical trial phase they normally worked with. Table 1 provides a summary of participant characteristics).

**Table 1:** Participant characteristics of 57 nurses and physicians in oncology/haematology

	Nurse (%) (***n*** = 31)	Physician (%) (***n*** = 26)	Total (%) (***n*** = 57)
**Country**			
Denmark	19 (61,3)	17 (65,4)	36 (63,1)
Sweden	12 (38,7)	9 (34,6)	21 (36,9)
**Sex**			
Female	29 (93,5)	18 (69,2)	47 (82,5)
Male	2 (6,5)	8 (30,8)	9 (17,5)
**Age**			
≤ 30–39	11 (35,8)	8 (30,8)	18 (33,3)
40–49	10 (32,1)	11 (42,3)	22 (38,6)
≤ 50–59	10 (32,1)	7 (26,9)	17 (29,7)
**Education**			
Bachelor (3 years)	22 (71,0)	–	22 (38,6)
Specialist nurses	9 (29,0)	–	9 (15,7)
Physicians (≥5 years)	–	9 (34,6)	9 (15,7)
PhD	–	17 (65,4)	17 (29,8)
**Years as nurse/physician**			
Mean	14,9 (0,3 to 35)	16,4 (1 to	15,5 (0,3 to
≤ 10	13 (42,0)	8 (30,7)	21 (36,0)
> 10	7 (22,5)	9 (34,6)	16 (28,0)
> 20	11(35,5)	9 (34,6)	20 (36,0)
**Years in oncology/ haematology**			
Mean	11,2 (0,3 to 35)	7,4 (1 to 40)	9,5 (0,3 to 40)
≤ 10	17 (54,8)	13 (50,0)	30 (52,6)
> 10	8 (25,8)	7 (27,0)	15 (26,3)
> 20	6 (19,3)	6 (23,0)	12 (21,1)
**Clinical trial setting**			
Phase I	13 (41,9)	11 (42,3)	24 (42,1)
Phase II	14 (41,1)	16 (61,5)	30 (52,6)
Phase III	27 (87,0)	24 (92,3)	51 (89,5)

A semi-structured interview guide was used covering three areas: 1) recruitment of patients in clinical trials; 2) ethical issues concerning recruitment to and working with clinical trial patients; and 3) potential strategies to address these ethical issues (Table 2). The issue specifically analysed in this article concerns the first and second areas, with focus on: a) advantages and disadvantages for the patient brought about by participation and b) whether or not the informants thought participating in a clinical trial was better for the patient. Informants provided informed written consent. The interviews, which were conducted in Swedish or Danish, lasted 30–70 min, were audio recorded and then transcribed verbatim by the authors or a research assistant. SP and ZEN conducted the interviews in Denmark and the informants in Sweden were interviewed by TG and an assistant.

**Table 2:** Interview guide

**I. Experiences when recruiting**	
• What does it mean for you to work with care and research? (opening question)	
• Can you describe how you inform patients about a research study?	
• What factors do you believe affect patients’ willingness to participate most?	
• Do you think patients understand the difference between medical care and research?	
**II. Ethical issues**	
• Have you felt on occasion that it would be unethical to include a patient, or ethical to exclude a patient?	
• Do you think there can be conflicts between personnel regarding care and research?	
• Have you met patients with unrealistic hopes for improvement?	
• Do you think patients want to participate in anything that they believe offers hope?	
**III. Strategies for dealing with dilemmas**	
• Do you discuss ethical issues in your workplace?	
• Do you remember how you or any colleague successfully dealt with one?	
• Do you feel that there are obstacles in your workplace that make it difficult to act ethically?	
• Is there something in your workplace that makes it easy to act ethically?	

##### Analysis of individual interviews

Inductive thematic analysis was used to examine the data [27]. After the first group of interviews, the researchers read the transcripts repeatedly to gain an initial understanding of the content. The group then met to discuss which codes to assign. Next, SE and ZEN took notes and coded the interview transcripts with physicians. ZEN, TG and SP did the interview transcripts with nurses, using the code “better for patients” for the relevant aspect. The resulting data were then further analysed in two separate sequences. First, ZEN did an inductive thematic analysis, which meant assigning units strongly linked to the data and corresponding to the aim of this study to more specific codes. Next, ZEN and TG grouped those codes into themes, and discussed the results with SE and MM.

This study adhered to the guidelines for empirical studies in Denmark and Sweden. No ethical approval was required because no sensitive personal data, as defined in Nordic guidelines, were used. Signed consent forms were collected before inclusion. Abbreviations (DP: Danish physicians, DN: Danish nurses, SP: Swedish physicians, and SN: Swedish nurses) were used to protect the identities of the nurses and physicians.

#### Part II: literature review

##### Conducting the review

ZEN searched PubMed for clinical trials comparing outcomes such as overall survival, progression-free survival and quality of life in cancer patients treated in and outside clinical trials. Because cancer treatment is undergoing rapid development, we sought to identify studies that included trials with patients enrolled within the last ten years. The following search terms were used: patients, controlled clinical, clinical trials, clinical trials phase I, II and III, disease-free survival, progression-free survival, survival analysis, quality of life, mortality, survival rate, treatment outcome, assessment, outcomes, trial effect, trial benefit, inclusion benefit, population outcome, survival, cancer patients, oncology and neoplasms. For full description of the search strategy, see Supplementary Material.

We screened all the studies by title, then by abstract, after which we read the full article, if at all relevant. The inclusion criteria were:

- Studies including a comparison of outcomes in cancer patients treated in and outside of pharmacological clinical trials
- Studies of trials that had been open for inclusion within the last 10 years, i.e. from January 2009 (if studies included more than one trial, at least one had to meet this criterion)
- Patients ≥18 years
- Primary research
- Studies written in Danish, English, Norwegian or Swedish

Exclusion criteria:

- Individual clinical trials which include other cancer treatment, in addition to pharmacological treatment (e.g. surgery and radiotherapy)
- Reviews and meta-analyses
- Studies including data from patients < 18 years of age, unless findings for that group were presented separately from ≥18 years of age
- Studies (i.e. research articles) that include trials other than pharmacological ones (e.g. intervention trials), unless data and findings for the pharmacological part were presented separately and thus possible to include
- Studies including trials with enrolment completed before January 2009, unless data and findings for trials with later enrolment were presented separately

##### Identification and review of research literature

**Fig. 2:**
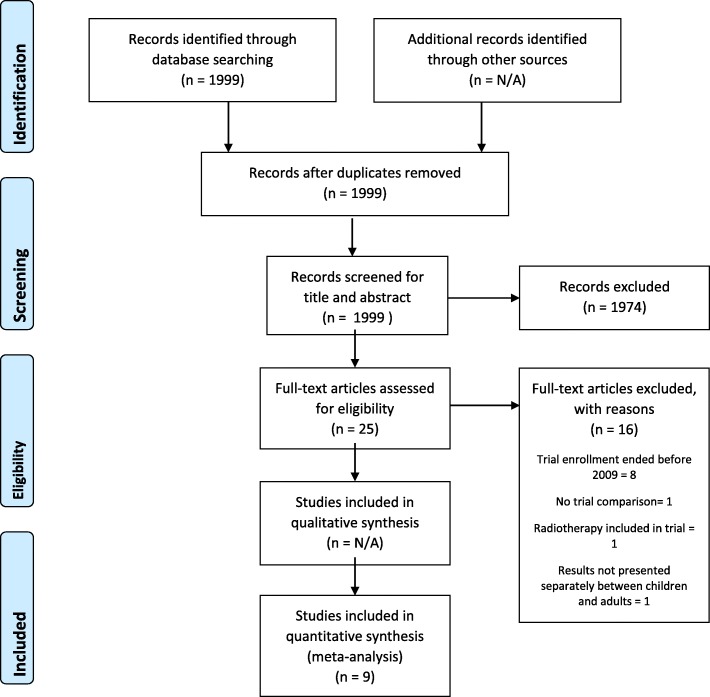
PRISMA Flow diagram showing the selection of articles

We identified nine eligible studies that included twelve comparisons of trial and non-trial patients: Arrieta et al. [28], Bertelli et al. [29], De Placido et al. [30], Field et al. [31], Goldman et al. [32], Khera et al. [33], Led Du et al. [34], Svensson et al. [35] and Templeton et al. [36]. Figure 2 contains a PRISMA flow diagram showing the selection of studies. Nine of the twelve comparisons were included in the review. One comparison was excluded because trial enrolment ended before 2009 (Templeton et al., [36]), while two others that included intervention trials were also excluded (Arrieta et al., [28]), in accordance with our exclusion criteria. Table 3 lists the comparisons that were included.

**Table 3:** Studies comparing cancer outcomes within and outside clinical trials

Reference	Population	Type	Dates	En-rolled^a^	Eligible controls	Treatment similarity^b^	Potential confounders and methods of control in adjusted analysis:^c^		Trial effect observed:^1^	
							Accounted for:	Unaccounted for:	Unadjusted:	Adjusted:^**d**^
1. Arrieta et al. [28]	Advanced non-small lung cancer	RC	2007–2014	143/889	No	No	**NBD:** Age, wood smoke exposure, exposure to asbestos, tobacco pack-years, brain metastases, ECOG PS, tobacco smoking group **MVA:** Disease stage, histology, sex, KRAS mutational status, EGFR mutational status, comorbidity^e^ **RoC:** Measurable disease, age > 18 years, ECOG PS ≤2 **SgPA:** Received chemotherapy^e^, positive EGFR mutation^e^	**NE:** SES **NA:** Treatment centre	Yes	Yes
2. Bertelli et al. [29]	Epithelial ovarian, fallopian tube, primary peritoneal cancer stage IV or sub-optimally debulked stage III	RC	2006–2010 (trial) 2012–2015 (real-world)	60/248	No	Yes	**RoC:** Patients managed by the South Wales Gynaecological Cancer Multidisciplinary Team, received at least one dose of bevacizumab as part of first-line treatment	**NA:** Sex **NE:** Stage, comorbidity, age, WHO PS, chemotherapy regimen	No	Not done
3. De Placido et al. [30]	HER2- positive metastatic breast cancer	RC	2008–2011 (trial) 2012–2015 (real-world)	155/402	No	Yes	**NBD:** Age, prior neoadjuvant therapy type **RoC:** Inclusion criteria: Received tratuzumab and pertu-zumab with taxane as first-line treatment for metastatic disease, no earlier RCT participation in a neo/adjuvant or metastatic setting **SgPA:** HR status, previously treated vs untreated with adjuvant and neoadjuvant tratuzumab, visceral vs non-visceral disease, > 2 metastatic sites vs 2–5 metastatic sites vs > 5 metastatic sites	**NE:** Comorbidity, PS, SES, treatment centre, sex **BD:** Brain metastasis, FISH / SISH / CISH statu	No	No
4. Field et al. [31]	Gliobla-stoma	RC	1998–2011	61/481	No	No	**MVA:** Age, IRSAD/SES, ECOG PS, operation type/biopsy **RoC:** Two co-located hospitals	**NE:** First-line clinical trial, comorbidity, tumour location, sex, multifocal disease, operation type, extent of resection, no. of operations, country of birth interpreter, residence, treatment centre, diabetes, smoking, tumour diameter **NA:** Stage	Yes	Yes
5. Goldman et al. [32]	Advanced stage melanoma unresecta-ble stage III or IV	PC	2006–2014	115/203	No	Yes	**MVA:** Sex, primary thickness, primary ulceration, ECOG PS, LDH (stage IV diagnosis), organs with metastases (treatment initiation), metastatic involvement **RoC:** Exclusion criteria: Treatments not administered by NYU oncologists, if records omitted pertinent data, patient lost to follow up, therapy administered adjuvantly **StrA:** Immunotherapy, targeted therapy	**NA:** Stage, treatment centre **SE:** SES, comorbidity **BD:** Age, prior systemic treatment	Yes	Yes
6. Khera et al. [33]	Acute leukaemia, myelo-dysplasia (MDS), chronic myeloid or myelo-monocytic leukaemia or myelo-fibrosis	RC	2004–2010	494/1353	Yes	Yes	**MVA:** Treatment, patient age at transplantation, diagnosis, disease risk, graft source, interval between diagnosis and HCT, KPS, HLA match, donor-recipient sex match, GVHD prophylaxis and use of ATG/alemtuzumab, regimen intensity, ethnicity, treatment centre^e^ **RoC:** Inclusion criteria: Age < 66 years, HCT. Exclusion criteria: Presence of donor-specific anti-HLA antibodies prior allogeneic or autologous transplantation < 12 months prior, HIV infection, pregnancy or breast-feeding, cardiac insufficiency or coronary artery disease requiring treatment, active infection, concomitant enrolment in a phase 1 study, a serum level of creatinine, bilirubin, ALT or AST that was greater than two times the upper limit of the normal range, forced vital capacity, forced expiratory volume in 1 s and/or diffusing capacity of the lung for carbon monoxide less than 50% of the predicted value **StrA:** Race, secondary leukaemia/MDS with prior auto, comorbidity, peripheral blood, bone marrow, sex (NBD)	**BD:** CMV match, donor age, year of transplantation **NE:** SES	No	No
7. Le Du et al. [34]	Metastatic breast cancer (MBC)	RC	2000–2010	285/367	Yes	No	**NBD:** PR status, radiation, age previous chemotherapy regimen **MVA:** Race, HER2 status, hormone receptor status, number of metastatic organs, previous neoadjuvant vs adjuvant chemotherapy, nuclear grade **RoC:** Sex, brain metastases, specific comorbidity, SES/treatment centre; patients living in Harris Country (MD Anderson location), eligible for a clinical trial according to usual inclusion and exclusion criteria for MBC trials and could benefit from the MD Anderson financial assistance programme support **SgPA:** ER positive, HER2 positive, triple negative breast cancer	**NE:** PS **BD:** Meta-static sites	No	No
8. Svensson et al. [35]	Metastatic castration-resistant prostate cancer (mCRPC)	RC	2008–2010 (trial) 2011–2016 (real-world)	119/1195	No	Yes	**NBD:** PSA, time since diagnosis at treatment start, Gleason score **RoC:** Inclusion criteria: male diagnosed with mCRPC, aged ≥18 years at time of treatment, treated for mCRPC with abiraterone	**NE:** PS, SES comorbidity, treatment centre **BD:** Age **NA:** Sex	No	Not done
9. Temple-ton et al. [36]	Metastatic castration-resistant prostate cancer	RC	2001–2012	43/314	No	Yes	**NBD:** Comorbidities, Gleason score, extent of disease: bones and visceral metastases, BMI, PSA-dt **MVA:** ECOG PS, year of first administration, haemoglobin, log LDH, log time from initial diagnosis, log PSA μg/l, baseline albumin, log ALP **RoC:** Exclusion criteria: Patients receiving weekly docetaxel, docetaxel in the context of neoadjuvant or adjuvant trials or as second-line chemotherapy	**NE:** SES **NA:** Sex, treatment centre **BD:** Age, extent of disease (lymph nodes)	Mixed^2^	No

*ALP* Alkaline phosphatases, *ALT* Alanine aminotransferases, *AST* Aspartate aminotransferases, *ATG* Antithymocyte globulin, *BD* Baseline Difference recorded, *BMI* Body mass index, *CISH* Chromogenic in situ hybridization, *CMV* Cytomegalovirus, *ECOG* Eastern Cooperative Oncology Group, *EGFR* Epidermal growth factor receptor, *ER* Oestrogen receptor, *FISH* Fluorescence in situ hybridization, *GVDH* Chronic graft-versus-host disease, *HCT* Hematopoietic cell transplantation, *HER2* Human epidermal growth factor receptor, *HIV* Human immunodeficiency virus, *HLA* Human leukocyte antigens, *HR* Hormone receptor, *IRSAD* Index of Relative Socioeconomic Advantage and Disadvantage, *KPS* Karnofsky performance score, *LHD* Lactate dehydrogenases, *MVA* MultiVariate/MultiVariable/MulticoVariate Analysis, *NA* Not Applicable, *NBD* No Baseline Difference, *NE* Not Evaluated, *NYU* New York University, *PC* Prospective Cohort, *PS* Performance status, *PR* Progesterone receptor, *PSA* Prostate specific antigen, *PSA-dt* Prostate specific antigen - doubling time, *RC* Retrospective Cohort, *RCT* Randomised controlled trial, *RoC* Restriction of cohort, *SES* Socioeconomic Status, *SISH* Silver in situ hybridization, *SgpA* Subgroup Analysis, *StrA* Stratified Analysis, *WHO* World Health Organisation

^a^ Values are number of participants in trial/non-trial group

^b^ Similarity between the treatment received by the trial participants and the treatment offered in the control group (for randomised controlled trials) and that was received by non-trial participants

^c^ We assessed whether each study attempted to account for possible confounding by age, sex (where applicable). PS, comorbidity, SES, stage (where applicable) and treatment centre

^d^ Adjusting for confounders by using multivariate or multivariable models, stratification, subgroup analysis, restriction of cohort

^e^ The methods section describes that this was adjusted in multivariate analysis but the results are not presented in the result section

^1^*p* < 0·05, unless otherwise noted

^**2**^ The Kaplan-Meier analyse showed a trial effect (*p* = 0.007), but the univariate Cox proportional hazard ratio did not (*p* = 0.089)

LBSH and ZEN analysed the included studies according to Peppercorn et al.’s conceptual framework [14]. First, the studies were summarised according to population, type of study, enrolment period, number of participants, eligible controls (yes/no, regarding whether controls met the same inclusion criteria as trial participants), treatment similarity, potential confounders and methods for control and trial effect observed. Similar to Peppercorn et al. [14], we assessed whether each study accounted for possible confounders by age, sex (if applicable), performance status, comorbidity, socioeconomic status, stage (if applicable) and treatment centre. Additional specific diagnostic factors were not assessed unless the authors of the studies mentioned them. The methods used for adjusting for confounders were multivariate/multivariable analysis, subgroup analysis, stratified analysis, restriction of cohort, assessment of baseline differences and matching of trial participants with non-trial participants (Table 3).

Next, the characteristics of the studies were categorised according to: design of trial versus non-trial comparison, type of clinical trial, type of malignant disease, baseline differences accounted for, type of analysis and non-trial patients restricted to those meeting trial eligibility criteria (Table 4). Lastly, the studies suggesting a trial effect were categorised according to type of malignant disease and type of study (Table 5).

**Table 4:** Characteristics of included studies (*n* = 9)

Design of trial versus non-trial comparison	
• Randomised controlled	0
• Natural experiment	0
• Eligible refuser	0
• Prospective cohort	1^a^
• Retrospective cohort	8^b^
Type of clinical trials patients participated in	
• Randomised only	**4**^**c**^
• Other	**5**^**d**^
Type of malignant disease	
• Haematological	**1**
• Solid tumour	**8**
Baseline differences accounted for	
• Age	**5**^**e**^
• Sex	**8**^**f**^
• Stage	**8**^**g**^
• Performance status	**5**^**h**^
• Comorbidity	**4**^**i**^
• Socioeconomic status	**4**^**j**^
• Treatment centre	**7**^**k**^
Type of analysis	
• Unadjusted only	**2**
• Adjusted only	**0**
• Both adjusted and unadjusted	**7**
Non-trial patients restricted to those meeting trial eligibility criteria	
• Yes	**2**^**l**^
• No	**7**

^a^ Goldman et al. [32]

^b^ Arrieta et al. [28], Bertelli et al. [29], De Placido et al. [30], Field et al. [31], Khera et al. [33] Le Du et al. [34], Svensson et al. [35], Templeton et al. [36]

^c^ All trials included in the four studies [29, 30, 33, 35] were phase III trials

^d^ In the five other studies, study design or trial phase could not be identified [28, 31, 32, 34, 36]

^e^ Three studies reported no baseline difference [28, 30, 34], and two studies adjusted using multivariate or multivariable analyses [31, 33]

^f^ Two studies adjusted using multivariate analysis [28, 32], one through restriction of cohort [34], and another did not identify any baseline difference [33]. For three studies, control of the confounder was not applicable [29, 35, 36]

^g^ One study explicitly adjusted for stage in the multivariate analysis [28], one study according to high vs low risk in multivariate analysis [33]. Four other studies in which the patients had metastatic disease at treatment start adjusted for, e.g. metastatic sites, prior treatment and specific mutations [30, 34–36]. Not applicable for two studies [31, 32]

^h^ Accounted for in multivariate or multivariable analyses [28, 31–33, 36]

^i^ One study accounted for this in a multivariate analysis as described in the method section, but the result is not presented [28], and another study by restriction of cohort [34]. Two studies did not identify any baseline difference [33, 36]

^j^ Two studies adjusted by restriction of cohort [31, 34], and two studies found no baseline difference [33, 36]

^k^ For three studies, adjusting for treatment centre was not applicable [28, 32, 36]. Three adjusted by restriction of cohort [29, 31, 34]. The last study did not investigate differences between the two included hospitals. One study adjusted through multivariable analysis [33]

^l^ Khera et al. [33], Le Du et al. [34]

**Table 5:** Studies indicating a trial effect

Type of malignant disease	
• Haematological [*n* = 1)	0
• Solid tumour (*n* = 8)	3^a^
Type of study	
• Prospective cohort (*n* = 1)	**1**^**b**^
• Retrospective cohort (*n* = 8)	**2**^**c**^
All studies (*n* = 9)	**3**

^a^ Arrieta et al. [28].], Field et al. [31], Goldman et al. [32]

^b^ Goldman et al. [32]

^c^ Arrieta et al. [28], Field et al. [31]

##### Characteristics of the studies

All nine studies included comparisons concerning overall survival [28–36], some also included progression-free survival [29, 30, 32, 34]. Eight of the nine studies were retrospective cohort studies. Four studies contained information on the design of included clinical trials, and they were all randomised phase III trials, which is the phase with the highest evidence for a possible treatment effect [37]; see Table 4 for a summary of study characteristics.

## Results

The results are presented in two parts. First, the results of the individual interviews with nurses and physicians are given and then the results of the literature review.

### Results from the interviews: how healthcare staff perceived the benefits of clinical trial participation

In this study, we asked physicians and nurses about how they perceived the advantages and disadvantages of participating in clinical trials for patients. We did not ask whether they regarded clinical trials as the best treatment option, but they often brought the subject up. Overall, they claimed that clinical trials offer patients the best treatment. In brief, the following sections will describe the general opinion of informants that the best treatment for cancer is in the context of a clinical trial, with physicians showing a greater belief in the benefit of participation than nurses. Sometimes informants expressed a steadfast conviction of direct trial effect while at other times their focus was on collateral benefits brought about by participation. The interviews also gave an opportunity for nurses and physicians to reflect upon the role of hope in trials and on the limitations of pursuing the trial option.

#### Clinical trials are important and better than standard care

Many physicians emphasised that only clinical trials that were expected to be at least as good as standard care would be accepted at their clinic. Otherwise, they were not permitted. One physician had the impression that the clinic would only choose to initiate trials if the treatment was better than standard care: “… when you agree to do a study, you should feel at ease, knowing that included patients are being given the best possible [treatment], so you are sure of that.” (SP9).

Some physicians were highly confident that clinical trials were the best treatment option for patients, even phase I trials. Consequently, they felt that it was important for patients to participate in trials: “… I believe it’s best for the patient. Also, because you get better results, whatever study you participate in, people say … I read that somewhere …” (SP7). One physician even saw it as unethical not to try to include patients in trials:

We’re supposed to give the best there is, and the one with the most evidence and … for many diagnoses we don’t know what’s the best possible treatment … then it’s almost unethical sometimes not to try to enroll patients in studies because, actually, we might otherwise be hurting the patient unnecessarily, when something basically better might be available … (SP9)

One physician said that lucky patients might get a treatment that is better than standard and believed that patients rarely receive treatment worse than standard, commenting that it might be so in theory but unusual in practice (SP4).

Some physicians believed that buying time through trial participation is beneficial for patients. Even if a trial might not provide a patient with a definitive improvement, it might then at least be possible to try something else later:

For me, it’s more important that they have the opportunity to receive an effective treatment and, concurrently, − how should I describe it? – that we buy time if the treatment is effective, but also that this presents us with more treatment options; that is, if you haven’t used the standard care options, you can always go back to them. Certainly, that’s an advantage. (DP7)

Similarly, some nurses held the belief that in the trials:

[You] may never make use of a protocol that’s not as good as the standard treatment … there must be a justified belief that [the trials] are as good as or better than the standard for us to, to conduct them, you know … (DN6)

These positive attitudes also appeared to be associated with the importance of doing studies for the benefit of future patients. The physicians highlighted the importance of conducting clinical trials to develop new and better treatment opportunities for future patients, and many physicians viewed it as their duty to recruit as many patients as possible to clinical trials – the more, the better.

I always say what I believe: “I’m pro-research, this is my view, you choose, you can always opt out of the study. This is the information.” If they don’t understand, I try to explain it again. Ehm … and then they can always return with their questions later, but I always tend to be on the positive side and include as many [patients]as possible. Because I believe that through more research, we may solve the riddle. (SP6)

While many informants talked about how they find clinical trials better than standard care, some of them struggled when trying to keep the balance between positive expectations about a trial and the fact that, in absence of trial evidence, there is no justification for believing it to be better. For example, when asked whether patients understood that it is impossible to know whether a trial will be better or worse than other treatments, one physician answered:

I really don’t know because I’m not even sure *we* understand this. Because we do the trial in the belief that it’s better, we find ourselves deeply challenged intellectually. Because if we didn’t believe it was better, we would have no reason to conduct it … and I’m obliged to tell them [the patients] that we are doing the trial because we hope it will be better, even though we don’t know if it is better until we’ve made a direct comparison. (DP1)

#### Various benefits

Many informants not only stated their belief that clinical trials can have a direct treatment effect for patients but also mentioned various collateral benefits brought about by participation. Closer follow-ups and monitoring may lead to various indirect positive effects. Thus, the assumption is that participants get a level of care that other patients might not have access to: “The greatest benefit, as I see it, is having contact with a research nurse, more controls, more tests being taken, scans and follow-ups on all their symptoms. Which all patients should have access to, really ...” (SP5).

A common viewpoint was that close follow-ups positively influenced the patients’ quality of life. One physician referred to quality of life studies illustrating that patients who received extra attention do better, regardless of the kind of examinations being undertaken:

We know that every time you read studies on quality of life, it’s always the participants who receive more attention of some kind that are better off. This is always the case. Regardless of which examinations and follow-ups were performed, those who get the attention usually benefit, so that can be an advantage, too. (DP3)

In general, physicians and nurses said that patients benefit from participation because they receive close attention from a permanent contact nurse and attend regular controls performed by a primary physician. Patients are also given the physician’s phone number, often the PI, which means a more experienced physician sees the patient. The main message was that patients, in their role as study participants, experience more and better continuity of care than patients in general: “… they will have personal contact with a very limited number of physicians [and be assigned] a contact nurse … so that results in a kind of care not available in standard care …” (SP2) and “They get more continuity” (DN12). Getting to know the carers can also promote trust, which is largely a positive experience: “I know they feel very secure” (DN12). A related aspect is safety. Several physicians said that closer follow-ups and fewer physicians around the patient made it easier for them to evaluate the effect of the study medicine. They felt that this could strengthen patients’ feelings of safety because the patients might then experience the treatment in the trial as being more similar to standard of care.

Lastly, several statements highlighted that having a larger number of patients included in a study can make it flow efficiently and productively. To receive patients at the reception, get them on the production line (as SP4 phrased it), and then doing follow-ups is a rational approach which might be beneficial for patients.

However, despite the widespread understanding that the close attention resulting from participation is beneficial, several physicians and many nurses stated that patient preferences determined whether frequent examination and monitoring was an advantage or a disadvantage. If patients considered frequent follow-ups and monitoring helpful, it actually was a benefit. Alternatively, if patients considered it a burden, it was a drawback.

Nurses often emphasised both the patients’ subjective judgement and the potential objective benefit of the study medicine, as judged by the medical staff: “Scans and some extra blood tests and visits to the doctor … well, it can be both [an advantage and a disadvantage]” (DN3). The same nurse further explained this by pointing out that this issue involves both what the patient wants and the actual outcome, i.e. whether or not something is advantageous depends on the patient, for some frequent appointments and contact are beneficial, but not for others. For example, undergoing a scan early in the process may not be beneficial due to the possibility of pseudoprogression, which may cause the patient to worry.

In this way, nurses pointed out that extra monitoring, scans and examinations are not always advantageous and sometimes become highly burdensome: “... this was, in any case, hard. I could see him losing weight and nearly failing to manage to get here for all his appointments. I can’t remember how it ended, but he died not long afterwards.” (DN17).

#### The role of hope in trial participation

The staff often stated that they, and their patients, too often put their hope in experimental phase I trials, especially patients at an advanced disease stage. This was often seen in immunotherapy, where patients put a great deal of hope in the prospect that the treatment, which aims to activate the immune response, would boost their life expectancy from months to years. Physicians and nurses often talked about offering hope in a difficult situation and stressed the usefulness of providing an alternative to palliative care: “In certain studies there’s probably over-confidence in the effect of a treatment, especially when it’s purely experimental ( …) In many cases the chances are slim ( …) Both the patient and ourselves would like to believe that it is more fruitful than it oftentimes is.” (SP8).

Some staff reflected upon whether putting too much hope into clinical trials might be a way for them to cope with patients having a poor prognosis:

… and I find working with a severe disease easier if a research project is involved. It creates a mental process that allows you to see … certain miserable things and you have the hope it will get better (…) the structure focuses not just on the highly tragic aspects but provides a framework that makes it easier to handle … the situation; that’s how it is for me. (SP2)

#### Limitations of trial participation

While the general belief was that trials are beneficial, more than a few informants presented a more nuanced assessment. Nurses typically described a higher degree of concern about patients being included in trials than physicians, who often based their hope on trials in the belief that there was a chance, however small, for patients to experience a therapeutic benefit from the study drug. Some physicians also reflected upon the uncertainties, potential risks and possibility that trials are less effective than standard care, as well as on trials failing to provide any significant results concerning progression-free survival. As one physician stated: “As healthcare staff, you know that the patient … if it’s a phase I trial, that the patient does not stand a chance” (SP11). Another physician brought up the effect of the low initial doses:

… usually, almost only homoeopathic doses are administered initially, and then you can allow the same patient to enter at a later stage, eh, when the dose goes up. Then you avoid the initial patients who may never benefit from the treatment, because it is more homoeopathy than, than proper treatment. (SP2)

Physicians were also aware of the fact that patients could receive a placebo instead of the experimental substance and thereby definitely not receive anything besides the standard treatment. However, these uncertainties were not often discussed among the colleagues:

“And the patients would rather not think about the experimental treatment possibly being worse, and really, I do not think we want to either. And we do not talk much about that.” (DP9).

Like these physicians, nurses distinguished between trial phases as there is a big difference between experimental phase I trials and randomised phase III trials. Nurses clearly showed less faith in the effectiveness of phase I trials. As one nurse said: “There are many ethical dilemmas. Yes. We talk about that a lot in particular with phase I trials, where the drugs are so novel that we strictly don’t know whether they work” (DN18). They acknowledged the possibility that the experimental treatment might do harm rather than good: “We experienced a great dilemma with those patients [in phase I] because we knew very well that it really wasn’t anything that could extend anyone’s life, rather the opposite” (DN9). Another nurse mentioned a particularly burdensome trial with little potential but with a risk of shortening lives: “… it prolonged life a couple of weeks and yet [we] sold the patients on this, even though they could get real sick, and I ... didn’t find it ethically correct to give it [the study drug] to them when it might actually shorten their lives.” (DN15).

Some of the nurses had such significant concerns about the lack of therapeutic effect or the use of placebo in clinical trials that they preferred patients to receive standard treatment instead. One possible reason is that they felt that patients could spend their final days in a better way, i.e. that the trial would be too burdensome. One nurse said: “And I think that it might be much better [for patients] to get what we can provide, which we know works, and then they only have to come here once every three weeks, not every week” (DN10). The very same nurse also highlighted that there are instances where waiting for an upcoming trial delayed treatment. If a study did not start for a few weeks, the nurses could not help but think to themselves: “You need treatment now!” The nurse then stated that when circumstances like this arise then patients should be told promptly.

## Results from the literature review: does clinical trial participation provide better outcomes for cancer patients?

### The empirical evidence of the study designs of the included studies

According to Peppercorn et al. [14] and Hariton et al. [38] we can distinguish between hierarchy of studies that graduates from those with the strongest emprical evidence and those with the weakest. At the top of the hierarchy is randomised controlled studies, the most suitable study design for investigating a trial effect and where the patient’s option to participate is randomised. Next is natural experiments or incidental randomisations, followed by trial participants being compared with eligible refusers, i.e. those who declined participation. Below that is prospective cohort studies and then, finally, retrospective cohort designs, which have important limitations due to the difficulty in controlling for baseline imbalances and the risk of hindsight bias. All nine of the studies included here fit in the lowest part of this hierarchy. One of the nine was a prospective cohort study [32] and the rest were retrospective cohort studies [28–31, 33–36].

Seven of the nine studies presented adjusted analysis for controlling of confounding effects or baseline differences [28, 30–34, 36] . The methods for adjusted analysis varied, including multivariable models [28, 31–34, 36], restriction of cohort [28, 30–34, 36], stratified- [32, 33] and subgroup analysis [28, 30, 34]. Only two trials restricted possible baseline confounders by restricting non-trial participants according to trial eligibility criteria [33, 34]. According to Peppercorn et al., it is essential that studies meet the above criteria to secure the validity of the results [14].

Whether pre-defined baseline characteristics were accounted for in the studies varied. Only half of the studies accounted for patients’ performance status, comorbidity and socioeconomic status in the adjusted analyses, and even then data were not available for all patients in some of studies [31–33], or the data were based on the researchers’ own interpretations [35]. The failure to account for baseline differences may be a result of conducting retrospective cohort studies for which it is not always possible to collect essential data due to a lack of reporting on non-trial patients [14].

As explained below, the variable character of what counts as “standard care”, used as a comparator in three studies, does not affect the results of each individual study or the results of our literature review. Arrieta et al. [28] conducted their study among a population at one Mexican hospital “Instituto Nacional de Cancerologia” and reports that all the included patients had received treatment according to the national and international guidelines. Field et al. [31] conducted their study on two hospitals (one public, one private) located in Victoria and reports that 1/3 of the included patients were treated at the private hospital. However, when discussing standard treatment, they refer to recognised Australian standards, so we are under the impression that the treatment regimens of the private and public hospitals are rather identical. Le Du et al. [34] compared trial participation with standard care. All the included patients in this study is from the MD Anderson Cancer Center in the USA, which is seated at the Texas Medical Center, a public hospital.

### Trial effect

Three of the nine comparisons suggested a trial effect, by which they refer to prolonged overall survival in both the unadjusted and adjusted part of their analysis [28, 31, 32]. The three studies suggesting a trial effect were all oncology studies, one used a prospective study design and the two others used a retrospective design. None of the three studies reported on which kind of trial (phases I-III) the trial participants participated in.

All three studies questioned their findings of a positive trial effect. Goldman et al.’s [32] prospective study asked whether the observed trial effect was caused by differences in baseline- and disease characteristics that were not included in the analysis. Studies by Arrieta et al. [28] and Field et al. [31] concluded that an experimental treatment effect may have caused the observed trial effect, but both studies had several limitations as they did not account for all the confounders included in the analytic framework for this review. Further, Field et al.’s [31] study had two important limitations. First, not all data for patients diagnosed before 2007 were available to the researchers. Second, no standard postoperative treatment was available for patients with glioblastoma prior to 2005, which may have contributed to the better survival rate in the trial participant cohort in the study [31].

Only two of the nine studies restricted non-trial participants to those meeting the inclusion criteria for the trial [33, 34], even though this is recommended to strengthen the validity of the retrospective study design [14]. These two trials did not observe a trial effect in their unadjusted and adjusted analyses [33, 34], but like all the other studies, they also did not account for all of the included confounders in the analytic framework for this review.

In summary, there is no strong evidence for the existence of a trial effect in cancer patients included in clinical pharmacological trials within the last ten years when compared with non-trial patients with either similar or non-similar treatment. There is some evidence for the absence of a trial effect, but these results are not based on adjusted analyses that account for all the selected confounders.

## Discussion

Our empirical interview results show a widespread belief among nurses and physicians that treatment for cancer in the context of a clinical trial is better than standard care. Many believe that recruiting patients to trials is a way to give them access to cutting-edge treatment, i.e. to provide them with access to the best treatment. As a result, some physicians felt a moral duty to recruit patients to clinical trials and thought that not doing so was unethical. Apart from such a direct experimental treatment effect, nurses and physicians also pointed out the possibility of a participation effect, where participation in the trial results in indirect but real positive effects. They acknowledged that such advantages could include more frequent monitoring and contact with healthcare staff.

We found that there is an overall belief among nurses and physicians that clinical trials provide a better treatment option than standard care, which is in line with previous results [39, 40]. Somkin et al. examined the involvement of oncologists in clinical trials and showed that the oncologists had “extremely favourable attitudes toward trials as a source of high-quality patient care” [41]. Furthermore, this belief is shared by the National Comprehensive Cancer Network (NCCN), which has the ambition to assist and offer guidelines to healthcare personnel, patients and their families. Their patient guidelines state: “Joining a clinical trial is strongly supported. NCCN believes that you will receive the best management in a clinical trial” ([42] p. 36).

Of course, there is an important difference between phase I, II and III trials. As the interviews were speaking about clinical trials in general and this is a retrospective study of a surprising find in the results, it is often not possible to determine whether an interviewee had only phase III studies in mind. When searching for evidence in the literature, it is a given that it would be phase III trials that stand the greatest chance of showing a trial effect. Their design is after all for that precise purpose. It is therefore of no consequence if some of the interviewees spoke primarily of phase I or II trials when claiming trials to be superior to standard care, as such a claim is even more unlikely to be true.

Our review of the research literature found no support for the belief that treatment in clinical trials is superior to standard care, despite the rapid development of cancer treatment in recent years, including use of targeted therapy and immunotherapy. Hence, our results, which are in line with older results by Peppercorn et al., showed that many studies did not control for relevant confounders and were biased. Lack of control of confounders and flaws in clinical trials are problematic, as is reporting bias, where benefits are systematically overstated, and disadvantages downplayed [43–46]. This lack of transparency might lead to a belief that trials have better results than is the case.

The interview material provided another way of understanding the belief in trial superiority. The informants mentioned various collateral benefits brought about by trial participation, such as more follow-ups and scans and more frequent contacts with the staff. However, as some nurses noted, this does not establish an objective superiority of trials over standard treatment. If these indirect effects (having more follow-ups, etc.) in turn would have had an overall positive biological effect, this would have impacted the results of the studies we have reviewed. When it comes to the subjective effects of frequent follow-ups, staff contact, etc., some patients might perceive that they are better off since they get more attention and feel taken care of, while others might perceive frequent hospital visits, tests, etc. in a negative vein. It remains unclear whether these indirect effects of study participation are overall perceived as dominatingly positive or negative.

In conclusion, our analysis of the literature does not support the belief that clinical trials provide the best treatment option. There is no support for general claims that trial participation is the best treatment option for the patient, and therefore it is essential that recruitment is not based on any claims or suggestions that it is. Doing so is an unjustified deviation from well-established requirements on the provision of information preceding adequate consent. Instead, patients should be encouraged to participate for the main purpose of making an altruistic contribution to the furthering of knowledge, to the potential benefit of future patients. When their sacrifice is great, as might be the case in phase I trials, maybe they would prefer to spend their last few days or weeks on something more beneficial to their own quality of life – but that choice should be theirs. Hence, making a decision based on hope is not always the best option.

### Strengths and limitations

Mixed methods has been suggested as a very good method for seeking to understand ethical aspects involved in clinical trials – and therefore explicitly encouraged [47]. Some of the strengths of the interview study are the large sample size (*n* = 57), the representativeness of the demographic characteristics, the variety of both short and extensive experience informants had with clinical trials from all trial three phases. Although clinical trials, like those we asked about, often are international, multicentre studies, this study was performed at academic trial centres at two university hospitals in Scandinavia and the results should therefore be interpreted with caution. It is possible that the results would have been different if physicians and nurses had been recruited from trial centres outside Scandinavian academic centres.

There are also some limitations to the literature review. First, a potential limitation concerns the exclusion criteria of omitting any clinical trial that included other cancer treatments besides pharmacological treatment. Including these trials could possibly have increased the number of studies examined here. Second, only one researcher (ZEN) searched the literature. However, in the planning stage of the project, a librarian (PS, see acknowledgement) supported us in developing the initial literature search strategy and in estimating the volume of relevant studies. Third, the results derive from only one database, PubMed. On the other hand, it should be noted that PubMed is the interface to MEDLINE, the world’s largest medical library [48].

Finally, note that while the literature review suggests that participation in trials does not lead to improved outcomes for patients, this does not serve as a basis for suggesting that participation in trials leads to poorer outcomes for patients. This would be to commit the inverse of the therapeutic misconception - the injurious misconception (see Snowdon et al. [49].^1^

## Conclusions

Many physicians and nurses make claims supporting trial superiority, the idea that being part of an experimental trial is always better than the standard treatment. We used Peppercorn et al.’s method to evaluate whether there is presently evidence available supporting that claim. Our study was unable to establish that trial treatment is superior to standard treatment. A weaker version of the claim is that trial participation causes indirect positive effects. However, as the actual value of such indirect effects is highly dependent on the individual’s specific circumstances and preferences, their existence cannot establish the general claim that treatment in trials is superior. Beliefs in trial superiority are therefore empirically unfounded. Hence, communicating these opinions to patients in a recruitment context is tantamount to providing misleading information. During recruitment, emphasis should instead be put on patients being able to make an altruistic contribution to furthering knowledge and to potentially benefitting future patients.

## Supplementary information

**Additional file 1.**

## Data Availability

The datasets used and analysed during the current study are available upon reasonable request.

## Abbreviations

ALP: Alkaline phosphatases
ALT: Alanine aminotransferases
AST: Aspartate aminotransferases
ATG: Antithymocyte globulin
BD: Baseline Difference recorded
BMI: Body mass index
CISH: Chromogenic in situ hybridization
CMV: Cytomegalovirus
ECOG: Eastern Cooperative Oncology Group
EGFR: Epidermal growth factor receptor
ER: Oestrogen receptor
FISH: Fluorescence in situ hybridization
GVDH: Chronic graft-versus-host disease
HCT: Hematopoietic cell transplantation
HER2: Human epidermal growth factor receptor
HIV: Human immunodeficiency virus
HLA: Human leukocyte antigens
HR: Hormone receptor
IRSAD: Index of Relative Socioeconomic Advantage and Disadvantage
KPS: Karnofsky performance score
LHD: Lactate dehydrogenases
MVA: MultiVariate/MultiVariable/MulticoVariate Analysis
NA: Not Applicable
NBD: No Baseline Difference
NE: Not Evaluated
NYU: New York University
PC: Prospective Cohort
PS: Performance status
PR: Progesterone receptor
PSA: Prostate specific antigen
PSA-dt: Prostate specific antigen - doubling time
RC: Retrospective Cohort
RCT: Randomised controlled trial
RoC: Restriction of cohort
SES: Socioeconomic Status
SISH: Silver in situ hybridization
SgpA: Subgroup Analysis
StrA: Stratified Analysis
WHO: World Health Organisation

## Footnotes

1 We gratefully acknowledge Bridget Young, who reviewed the paper, for this point.
