# Supplementary material for: Are cancer patients better off if they participate in clinical trials? A mixed methods study
Source: BMC Cancer. 2020 May 8;20:401. doi: 10.1186/s12885-020-06916-z (PMC7206768; doi:10.1186/s12885-020-06916-z)
Supplement: Supplementary file 1 — Additional file 1. [file 12885_2020_6916_MOESM1_ESM.docx]

| Search | Search term | Results |
| --- | --- | --- |
| #39 | #38 Filters: Full text; published in the last 10 years; Humans; Danish; English; Norwegian; Swedish; Adult: 19+ years | [1999](https://www.ncbi.nlm.nih.gov/pubmed/?cmd=HistorySearch&querykey=121) |
| #38 | #37 Filters: Full text; published in the last 10 years; Humans; Danish; English; Norwegian; Swedish | 3416 |
| #37 | #36 Filters: Full text; published in the last 10 years; Danish; English; Norwegian; Swedish | 3424 |
| #36 | #35 Filters: Full text; published in the last 10 years; Danish; English; Norwegian | 3424 |
| #35 | #34 Filters: Full text; published in the last 10 years; Danish; English | [3424](https://www.ncbi.nlm.nih.gov/pubmed/?cmd=HistorySearch&querykey=117) |
| #34 | #33 Filters: Full text; published in the last 10 years; Danish | 0 |
| #33 | #32 Filters: Full text; published in the last 10 years | [3510](https://www.ncbi.nlm.nih.gov/pubmed/?cmd=HistorySearch&querykey=114) |
| #32 | #31 Filters: Full text | [6050](https://www.ncbi.nlm.nih.gov/pubmed/?cmd=HistorySearch&querykey=113) |
| #31 | #30 NOT #29 | [7500](https://www.ncbi.nlm.nih.gov/pubmed/?cmd=HistorySearch&querykey=112) |
| #30 | #27 NOT #28 | [8731](https://www.ncbi.nlm.nih.gov/pubmed/?cmd=HistorySearch&querykey=111) |
| #29 | meta analysis [Publication Type] | [99132](https://www.ncbi.nlm.nih.gov/pubmed/?cmd=HistorySearch&querykey=88) |
| #28 | review [Publication Type] | [2502296](https://www.ncbi.nlm.nih.gov/pubmed/?cmd=HistorySearch&querykey=87) |
| #27 | #22 AND #26 | [19477](https://www.ncbi.nlm.nih.gov/pubmed/?cmd=HistorySearch&querykey=110) |
| #26 | #24 OR #25 | [3342017](https://www.ncbi.nlm.nih.gov/pubmed/?cmd=HistorySearch&querykey=109) |
| #25 | cancer patients | [1252363](https://www.ncbi.nlm.nih.gov/pubmed/?cmd=HistorySearch&querykey=108) |
| #24 | "neoplasms"[MeSH Terms] | [3151714](https://www.ncbi.nlm.nih.gov/pubmed/?cmd=HistorySearch&querykey=85) |
| #23 | #8 AND #22 | [69262](https://www.ncbi.nlm.nih.gov/pubmed/?cmd=HistorySearch&querykey=84) |
| #22 | #9 OR #10 OR #11 OR #12 OR #13 OR #14 OR #15 OR#16 OR #17 OR #18 OR #19 OR #20 OR #21 | [1924718](https://www.ncbi.nlm.nih.gov/pubmed/?cmd=HistorySearch&querykey=83) |
| #21 | survival [MeSH Terms] | [4569](https://www.ncbi.nlm.nih.gov/pubmed/?cmd=HistorySearch&querykey=49) |
| #20 | population outcome | [174842](https://www.ncbi.nlm.nih.gov/pubmed/?cmd=HistorySearch&querykey=47) |
| #19 | inclusion benefit | [11752](https://www.ncbi.nlm.nih.gov/pubmed/?cmd=HistorySearch&querykey=46) |
| #18 | trial benefit | [88287](https://www.ncbi.nlm.nih.gov/pubmed/?cmd=HistorySearch&querykey=45) |
| #17 | trial effect | [296474](https://www.ncbi.nlm.nih.gov/pubmed/?cmd=HistorySearch&querykey=44) |
| #16 | assessment, outcomes [MeSH Terms] | [991709](https://www.ncbi.nlm.nih.gov/pubmed/?cmd=HistorySearch&querykey=43) |
| #15 | treatment outcome [MeSH Terms] | [964720](https://www.ncbi.nlm.nih.gov/pubmed/?cmd=HistorySearch&querykey=42) |
| #14 | quality of life [MeSH Terms] | [173843](https://www.ncbi.nlm.nih.gov/pubmed/?cmd=HistorySearch&querykey=41) |
| #13 | survival rate [MeSH Terms] | [160995](https://www.ncbi.nlm.nih.gov/pubmed/?cmd=HistorySearch&querykey=40) |
| #12 | mortality[MeSH Terms] | [356656](https://www.ncbi.nlm.nih.gov/pubmed/?cmd=HistorySearch&querykey=39) |
| #11 | analyses, survival [MeSH Terms] | [270653](https://www.ncbi.nlm.nih.gov/pubmed/?cmd=HistorySearch&querykey=38) |
| #10 | progression free survival [MeSH Terms] | [530](https://www.ncbi.nlm.nih.gov/pubmed/?cmd=HistorySearch&querykey=37) |
| #9 | disease free survival [MeSH Terms] | [68613](https://www.ncbi.nlm.nih.gov/pubmed/?cmd=HistorySearch&querykey=36) |
| #8 | #1 AND #7 | [133608](https://www.ncbi.nlm.nih.gov/pubmed/?cmd=HistorySearch&querykey=35) |
| #7 | #2 OR #3 OR #4 OR #5 OR #6 | [323925](https://www.ncbi.nlm.nih.gov/pubmed/?cmd=HistorySearch&querykey=34) |
| #6 | clinical trials, phase iii as topic" [MeSH Terms] | [8611](https://www.ncbi.nlm.nih.gov/pubmed/?cmd=HistorySearch&querykey=33) |
| #5 | Search clinical trials, phase ii as topic [MeSH Terms] | [7266](https://www.ncbi.nlm.nih.gov/pubmed/?cmd=HistorySearch&querykey=32) |
| #4 | clinical trials, phase i as topic [MeSH Terms] | [5049](https://www.ncbi.nlm.nih.gov/pubmed/?cmd=HistorySearch&querykey=31) |
| #3 | clinical trials as topic [MeSH Terms] | [129968](https://www.ncbi.nlm.nih.gov/pubmed/?cmd=HistorySearch&querykey=29) |
| #2 | clinical trials, controlled as topic [MeSH Terms] | [129968](https://www.ncbi.nlm.nih.gov/pubmed/?cmd=HistorySearch&querykey=29) |
| #1 | patients [Title/Abstract] | [5319712](https://www.ncbi.nlm.nih.gov/pubmed/?cmd=HistorySearch&querykey=28) |
